# The clinical effect and safety of the treatment of tibia intercondylar eminence fracture with cannulated screw and suture fixation under arthroscope: Protocol for a systematic review and meta-analysis of randomized controlled trials

**DOI:** 10.1097/MD.0000000000020609

**Published:** 2020-06-05

**Authors:** Peng Xu, Lu-Chang Liu, Qi-Jun Chen, Ping Yang, Xiao-Bin Chen, Xiao-Ping Xie

**Affiliations:** a First Department of Orthopedics; b Department of Stomatology, The Second People's Hospital of Yibin, Cuiping, Yibin; c Department of Pathology, West China Second University Hospital, Sichuan University, Wuhou; d Department of Gastrointestinal Surgery, Sichuan Academy of Medical Sciences & Sichuan Provincial People's Hospital (Eastern Hospital), Chengdu; e Department of Urology, The Second People's Hospital of Yibin, Yibin, Sichuan, China.

**Keywords:** arthroscopy, cannulated screw fixation, clinical efficacy, meta-analysis, randomized controlled trial, suture fixation, tibia intercondylar eminence fracture

## Abstract

**Background:**

The clinical effects and safety over the treatment of tibia intercondylar eminence fracture (TIEF) with cannulated screw and suture fixation were evaluated under arthroscope systematically, providing evidence-based medical support for the selection of surgical methods in terms of minimally invasive arthroscopic treatment for TIEF.

**Methods:**

The English databases of PubMed, EMBASE, Cochrane Library, CNKI, SinoMed, VIP, and Wanfang databases were searched by computer. The randomized controlled trials were conducted to compare the clinical effects of TIEF with cannulated screw and suture fixation under arthroscope. The retrieval period is from the beginning of database building to January 2020. There is no language restriction. Chinese databases are searched by keywords, while English databases are searched by the combination of subject words and free words. According to the retrieval strategy, the two evaluators will lead the conforming documents into Note Express for repeated literature screening, and the two evaluators will extract and cross-check the conforming documents according to the pre-designed data extraction table. Two researchers adopted the modified Jadad scale independently to evaluate the quality of the literature. The RevMan 5.3 version software provided by the Cochrane Collaboration Network was adopted for statistical analysis.

**Results:**

The study will strictly review and extract the data included in the literature, and scientifically make statistical analysis for the pre-set outcome indicators. All the research processes will be conducted in strict accordance with the guidance of system evaluation. In this study, the differences between cannulated screw fixation and suture fixation under arthroscopy will be evaluated by comparing the relevant outcome indicators. All the results of this study will be published openly in a highly influential professional academic journal.

**Conclusion:**

The paper adopted Cochrane system evaluation method to collect and sort out the published literature about the treatment of tibial eminence fracture between cannulated screw fixation and suture fixation under arthroscopy, and to compare the clinical efficacy and safety of the two fixation methods utilizing meta-analysis and comparison of related outcome indicators. Through this study, we will draw a positive conclusion, which will provide a basis for the better treatment of tibial eminence fracture.

**PROSPERO registration number:**

PROSPERO CRD42020168433.

## 1 Introduction

Tibia intercondylar eminence fracture (TIEF), which is also known as intercondylar eminence fractures, are a special type of intra-articular fractures. Scholar Poncet is the one who first described the breach of TIEF in 1875.^[1,2]^ In the early stage, this kind of breach is commonly seen among the children and teenagers who are injured by transportation, such as, bicycle or motorcycle, and so on. while the incidence of this kind of fracture is relatively low. However, with the growth of people's enthusiasm for sports and the development of the social transportation industry, the rise of various sports injuries and traffic accidents, intercondylar eminence fractures in adults has also become a commonly seen knee joint injury with the increasing incidence. It is reported that there are 3 cases of intercondylar eminence fractures among every one hundred thousand people every year on average.^[3,4]^ As the anterior cruciate ligament (ACL) starts from the anteromedial slope of tibial eminence fracture, when the tibial eminence is fractured, it will often lead to the change of the starting point position of ACL, which will inevitably lead to the relaxation of ACL tension, and various complications such as knee traumatic arthritis or meniscus injury will be caused by further development. What's more, ACL also plays an important effect on the forward stability of the knee joint, limiting the excessive forward movement of the tibia, and plays a familiar role in stabilizing the knee joint with the lateral collateral ligament and the posterior capsule of the knee joint. When this fracture occurs, the ACL stability of the knee joint is damaged, resulting in ACL relaxation, abnormal elevation of avulsed bone mass, which leads to the impact of the intercondylar fossa, and then the joint instability. If not treated in time, other tissues in the knee joint such as bone, cartilage, ligament, and meniscus will be damaged, besides, avulsion fracture of tibial intercondylar crest in adults is often associated with meniscus injury and ACL injury, and so on, leading to the occurrence of free body of articular cartilage, impact of intercondylar fossa or instability of knee joint. Therefore, restoring the continuity and tension of ACL is one of the primary purposes of fracture reduction and internal fixation.^[5–7]^

The classification over the most common for TIEF is proposed by Meyers and Mc Keever, and so on^[8]^ and they divided it into three types: the first type is the fracture without displacement or minimum displacement; the second type is the fracture with avulsed bone block tilted from 1/3 to 2/3 of the front with no displacement fracture at the back; the third type is the avulsion fracture with complete displacement. The third type of fractures can be further divided into two types: The first type of Type III involves only the ACL attachment area and the second type of Type III involves the entire intercondylar eminence. Some people have also labeled comminuted fractures as type IV. At present, conservative treatment is recommended for type I fracture, at the same time, surgical intervention is generally required for fractures of type II and III, mainly including open reduction and internal fixation and arthroscopic reduction and internal fixation (ARIF). Open reduction and internal fixation is a relatively ancient operation method, which cut the exposed fracture surface directly, conducting the reduction and fixation of fracture under direct vision. However, it has a large surgical incision, causing significant damage to the surrounding soft tissue, muscle and intra-articular structure with long pain duration and recovery time after operation, and may cause muscle contracture around the knee mutual, stiff knee joint adhesion, and even arthritis in the later stage, so that it has been gradually replaced by ARIF.^[9,10]^ ARIF is the abbreviation of ARIF, which is the latest surgical method for the treatment of TIEF, which was carried out by McLennan in 1982 for the first time.^[11]^ For the treatment of TIEF, ARIF is an effective method, significantly reducing the stay length and pain duration. Generally, ARIF needs two surgical approaches for operation. One is the observation approach, which is used for arthroscopy, the other is an operation approach, which is used for fracture reduction and fixation. Sometimes, due to the complexity of the breach, difficult the fracture reduction is difficult, and a small incision can be taken in the middle of the lower patellar tendon. All in all, with the technology becoming more and more mature, fixed methods are also developing.

At present, hollow screw fixation and suture fixation are the most widely used and reliable surgical techniques, but the advantages and disadvantages of these two fixation methods are still controversial among clinicians.^[12,13]^ Therefore, this study systematically evaluates the clinical efficacy and safety of hollow screw and suture fixation under arthroscope in the treatment of TIEF by means of meta-analysis, providing evidence-based medical support for arthroscopic minimally invasive treatment of TIEF.

## 2 Materials and methods

### 2.1 Registration

This systematic evaluation has been registered in PROSPERO (https://www.crd.york.ac.uk/PROSPERO/). Registration Number: CRD42020168433.

### 2.2 Study inclusion and exclusion criteria

#### 2.2.1 Types of studies

Only randomized controlled trial was included in the study, involving literature on arthroscopic fixation of tibial intercondylar eminence fracture with cannulated screw and suture without language restrictions, and without restrictions on the time and form of publication of the literature.

#### 2.2.2 Types of participants

The participants met the diagnostic criteria of TIEF. At the same time, arthroscopic cannulated screw fixation or suture fixation can be used for TIEF.

##### 2.2.2.1 Inclusion criteria

(1) All the literatures are randomized controlled trials (RCT);
(2) patients diagnosed as fracture of tibial intercondylar eminence fracture and rupture of ACL;
(3) patients with normal limb function before injury;
(4) patients with good compliance, who can follow the doctor's advice for rehabilitation exercise and regular follow-up.
(5) Both the experimental group and the control group were set up in the literature, including the patients fixed with hollow screw and suture in both groups.
(6) There were outcome indicators with consistent judgment criteria;
(7) complete data.

##### 2.2.2.2 Exclusion criteria

(1) defects or errors in the design of the study plan;
(2) the original literature was non-randomized controlled trial;
(3) the study was a summerized, case report, animal experiment;
(4) short follow-up time with the high rate of loss.
(5) The affected limb belongs to open fracture, multiple fracture or tibial plateau fracture; the meniscus tear, anterior and posterior cruciate ligament and other ligaments of knee joint of the affected limb are seriously injured;
(6) the patient is combined with important organ injury, such as, brain, chest, and abdomen;
(7) the patient is combined with heart, liver, kidney and other important organ dysfunction or other serious diseases.

#### 2.2.3 Types of interventions

##### 2.2.3.1 Control group

The tibial intercondylar eminence fracture was treated by arthroscopic suture fixation.

##### 2.2.3.2 Experimental group

The tibial intercondylar eminence fracture was treated by arthroscopic cannulated screws.

### 2.3 Types of outcomes.

#### 2.3.1 Main outcome indicators

Postoperative knee stability score:^[14,15]^

(1) Lachman test;
(2) KT-1000.

Postoperative knee function score:^[16–18]^

(1) Tegner score;
(2) Lysholm score;
(3) IKDC 2000 subjective score of knee function;
(4) knee mobility.

#### 2.3.2 Secondary outcomes

(1) Operation duration;
(2) Intraoperative bleeding volume;
(3) Hospital stay;
(4) Complications;
(5) Treatment costs

### 2.4 Searching strategy

The English databases including PubMed, EMBASE, Cochrane Library, Chinese databases CNKI, SinoMed, VIP, and Wanfang databases were searched by computer. The RCT were conducted so as to compare the clinical effects over arthroscopic fixation of tibial intercondylar eminence fracture with cannulated screw and suture. The retrieval period is both from the beginning of the establishment of each database to January 2020 without language restrictions. Meanwhile, the retrieval references included in the research can improve the recall ratio of literature. The Chinese database uses common words for recovery; the English database uses the combination of subject words and free concepts for retrieval. The retrieval strategy for PubMed is shown in Table 1.

**Table 1:**
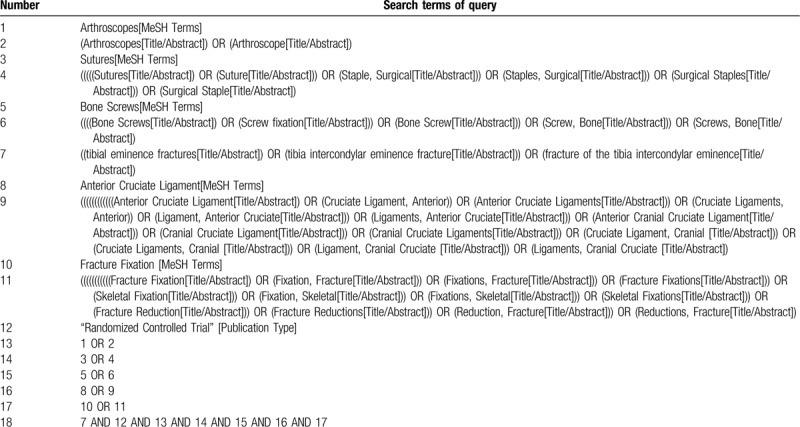
Search strategy used in PubMed database.

### 2.5 Data collection and analysis

**Figure 1:**
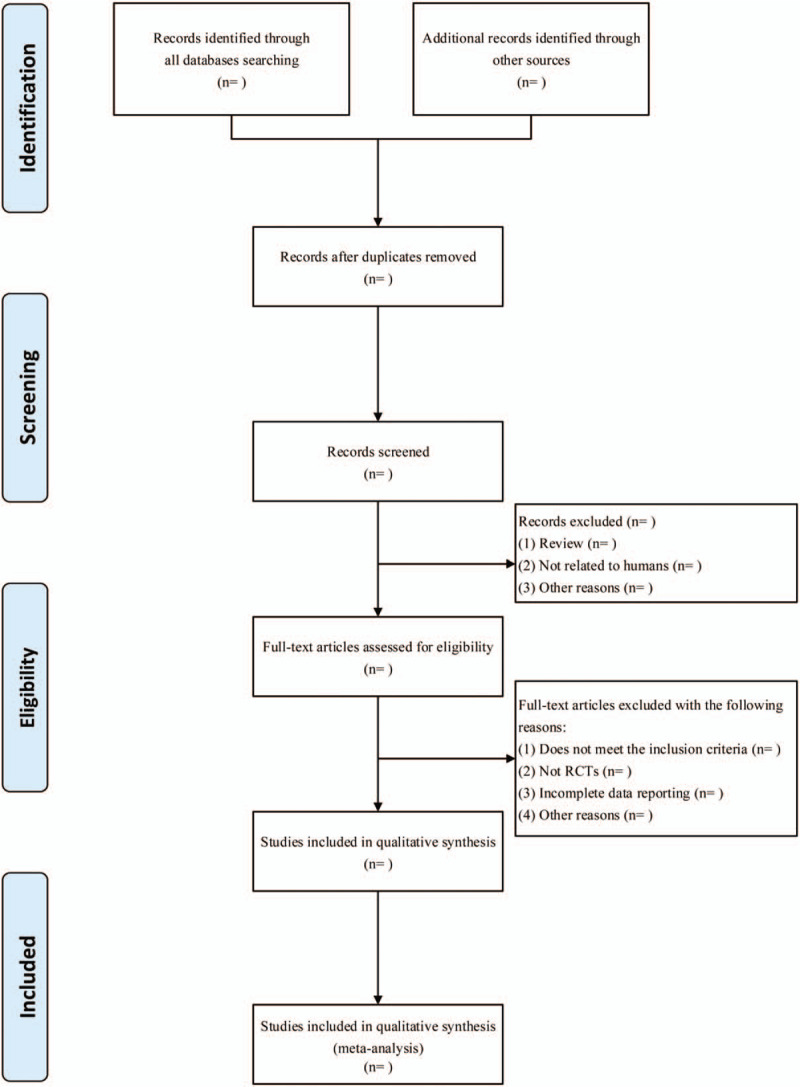
Flow chart of the PRISMA research selection.

The quality evaluation criteria were selected, extracted, evaluated and cross-checked by 2 researchers independently in strict accordance with the inclusion criteria and exclusion criteria. After reading the title and abstract of each document, each researcher reads the title and abstract of each document and conducts preliminary screening. Intensive reading can be included in the full text of the meta-analysis literature. In the process of literature evaluation, if two researchers have different opinions on literature evaluation, a third researcher can be added, and the opinions can be unified through intra group research and discussion. The quality of each literature is evaluated and the collected relevant data is cross-checked. For the relevant research literature with insufficient data, the contact with the author of the literature and the person in charge of the clinical trial will be served as a supplement and the differences can be resolved through discussion. The flow chart of the Preferred Reporting Requirements for Systematic Review and Meta-Analysis Protocol research selection is shown in Figure 1.

### 2.6 Assessment of risk of bias

According to the system evaluation manual provided by Cochrane, the improved Jadad score scale for quality scoring was adopted with the article carefully read. The scores of randomized RCT method, the implementation of the blind method, follow-up, withdrawal, and so on, were evaluated and scored respectively with the quality score of the whole passage obtained finally. The improved Jadad score scale is 7 points. The article with low quality is more than or equal to one point and less than or equal to three points and the article with high quality is more than or equal to 4 points.^[19]^

### 2.7 Statistical analysis

#### 2.7.1 Measures of treatment effect

Meta-analysis used RevMan 5.3 software. Risk ratio was used for the statistical data and mean difference were used for a continuous variable, and 95% CI of both were used for the statistic of curative effect analysis.

#### 2.7.2 Assessment of heterogeneity

The *X*^2^ was used to test and analyze statistical heterogeneity with no clinical heterogeneity (*P* > .1 or I^2^<50%). The fixed effect model was used for measuring results to conduct meta-analysis; When there is statistical heterogeneity (*P* < .1, *I*^2^ > 50%), the source of heterogeneity should be tried to find out. For further subgroup analysis or sensitivity analysis, if the heterogeneity still exists, then the random effect model for meta-analysis should be used at this time; when there is apparent clinical and statistical heterogeneity between the research results, then the descriptive analysis should be applied.

#### 2.7.3 Sensitivity analysis and subgroup analysis

Sensitivity analysis refers to observing the incidence of a specific or a group of factors by changing a certain research factor in a meta-analysis. In this paper, RevMan 5.3 software is used to analyze the sensitivity of the included literature through the transformation effect model method or impact analysis method to verify the reliability for the results of meta-analysis. The transformation model method refers to when the mutual transformation occurs from the fixed-effect model or random-effect model, and the results do not change substantially. It can be considered that the results of the meta-analysis are reliable. Otherwise, it is believed that the results are not safe. The impact analysis method refers to remove the included literature one by one to observe whether the heterogeneity of meta-analysis has substantive changes to determine the source of heterogeneity.

In this study, subgroup analysis was carried out according to the following classification when possible.^[20]^

(1) Study on the subgroup analysis for low and high risk of bias
(2) Subgroup analysis was conducted according to the course of disease, operation time and age range of patients
(3) Subgroup analysis was conducted according to the length of follow-up time

### 2.8 Publication bias

A funnel chart is usually used to test and analyze publication bias in research. Small samples are generally at the bottom of funnel chart, because the dispersion is relatively large; on the contrary, large examples are generally at the top because of the relatively small distribution.

In general, the funnel chart, should be large at the bottom and small at the top if there is no bias. If not, there may be a relatively significant bias, which may be caused by publication bias. If the funnel diagram is symmetrical, there is no publication bias. If the funnel diagram is asymmetric or incomplete, it indicates that there may be publication bias.^[21]^

### 2.9 Ethics and dissemination

Clinical approval is not applicable for this study because the type of this study is a systematic review.

## 3 Discussion

The TIEF the attachment point of tibial end of ACL of the knee joint. Once the breach occurs due to trauma, ACL of the knee joint is bound to be damaged, and the ACL of the knee joint plays an essential role in maintaining the stability of knee joint. If avulsion of tibial intercondylar ridge is not adequately reduced or fixed, it will directly lead to the relaxation and dysfunction of ACL, directly affect the stability of knee joint, and even accelerate the progression of knee osteoarthritis. Regarding the treatment of TIEF, the specific treatment method should be selected according to the classification, and severity of the breach. For type I fracture, conservative treatment is recommended. An adjustable brace can be used to fix the knee joint in the neutral position for 4 to 6 weeks. Plaster fixation of the lower limbs is not recommended. As for fractures of type II and III, surgical treatment is required. There are two main operative methods for TIEF, that is, open reduction and internal fixation and ARIF. Compared to arthroscopic minimally invasive surgery, open reduction and internal fixation have more disadvantages and complications, including soft tissue injury, more prolonged postoperative pain, and hospital stay.^[22]^

With the development of technology and equipment in recent years, arthroscopic minimally invasive surgery has gradually replaced the traditional arthrotomy in the treatment of TIEF. Arthroscopic minimally invasive technique has become the gold standard for the treatment of TIEF, because they can better observe the intra-articular injury, simplify the diagnosis, and handle breach accurately. Besides, it is easier to remove the loose bone fragments and deal with the related soft tissue injury. Arthroscopic treatment should first clear the surface of the avulsed bone and remove the inserted soft tissue, because it may cause extrusion and make it difficult to anatomical reduction.^[23]^ Moreover, Implant fixation of fracture block may then be considered for fixation of the fracture mass.

At present, the main controversy in clinical practice is about which fixation method should be used. It is reported that screw and suture fixation is the most widely used and reliable surgical technique at home and abroad. But at present, there is still lack of evidence-based medicine. Through this study, we will draw a positive conclusion, which can provide a basis for better clinical treatment of TIEF.

## Author contributions

**Conceptualization:** Xiao-Ping Xie, Peng Xu.

**Data curation:** Xiao-Ping Xie, Lu-Chang Liu.

**Formal analysis:** Peng Xu, Lu-Chang Liu.

**Methodology:** Qi-Jun Chen, Ping Yang, Xiao-Bin Chen

**Software:** Peng Xu, Qi-Jun Chen.

**Supervision:** Peng Xu, Lu-Chang Liu

**Writing – original draft:** Peng Xu, Lu-Chang Liu, Qi-Jun Chen, Ping Yang, Xiao-Bin Chen.

**Writing – review & editing:** Xiao-Ping Xie.
